# Turkish Validity and Reliability Study of Challenge-Hindrance Demands Scale for the Nursing Profession

**DOI:** 10.1155/2022/3912003

**Published:** 2022-09-22

**Authors:** Handan Ertaş, Emel Fi̇li̇z, Şeyma Kahveci̇, Seda Nur Ünal

**Affiliations:** ^1^Selcuk University Faculty of Health Sciences, Konya, Turkey; ^2^KTO Karatay University Vocational School of Health Services, Konya, Turkey

## Abstract

This study aims to adapt “the Challenge-Hindrance Demands Scale for Nursing Professional” developed by Mahaverachartkul and Sooraksa; regarding “how nurses who are under heavy workload and stress as well as intense and tiring working hours perceive many stressful situations in the working environment as challenges or hindrances” to Turkish language and bring it to the use of national literature. In terms of method, the research was designed within the scope of the quantitative research model. Data collected from 450 nurses were used in this study. The research data were analyzed in SPSS 26.0 and AMOS 24.0 statistical programs. The validity of the scales used in this study was evaluated with exploratory factor analysis (EFA) and confirmatory factor analysis (CFA). According to the EFA findings of the Challenge Demands Scale, when the KMO value (0.919) and the Bartlett sphericity test value (X2 (78) = 4121.203; *p*=0.001) were examined, it was seen that the data were suitable for analysis. When the CFA findings of the Challenge Demands Scale were examined, it was seen that the fit indices were at an acceptable level (X2 (60) = 223.912; *p*=0.001; CMIN/df = 3.732; CFI = 0.960; TLI = 0.948; RMSEA = 0.078; SRMR = 0.039). According to the EFA findings of the Hindrance Demands Scale, when the KMO value (0.947) and the Bartlett sphericity test value (X2 (78) = 5263.056; *p*=0.001) were examined, it was seen that the data were suitable for analysis. When the CFA findings of the Hindrance Demands Scale were examined, it was seen that the fit indices were at an acceptable level (X2 (61) = 208.794; *p*=0.001; CMIN/df = 3.423; CFI = 0.972; TLI = 0.964; RMSEA = 0.073; SRMR = 0.032). In conclusion, the Challenge-Hindrance Demands Scale for Nursing Professionals with 13 items and four dimensions developed to measure the stress, hindrance, and challenge levels of nurses in their professional life in Turkey may be used in future studies.

## 1. Introduction

Human resources are central to managing and delivering health services worldwide [1]. The ability of a country's health systems to perform well and respond appropriately to new challenges is strongly influenced by the presence of a sufficient number of health professionals with relevant skills in an environment where they are needed, and that motivates them [2]. For this reason, health institutions become service organizations that host many occupational groups. The group with the most comprehensive workload among these occupational groups is considered to be nurses [3]. In the Nursing Report published by the World Health Organization, it was estimated that there are 27.9 million nurses in 191 countries. The report emphasized that this number represents more than half of all health professionals [4]. According to Reference [5] data, nurses constituted 15% of the health workforce in Turkey, and there were 171,259 nurses in Turkey.

Moreover, the number of nurses per 100,000 people in 2020 was 342 [5]. In other words, there are 3.4 nurses per 1,000 people. In 2018, the number of nurses per 1,000 people in 36 OECD countries was 8.8. In the world, the number of nurses per 1.00 people was calculated as 3.6 [6]. This indicates that nurses who devotedly provide the services for protecting and improving individual and public health, provide patient care with scientific methods, serve society by adopting the principle “humans first”, and fulfill an important and sacred duty for human health represent a crucial occupational group for every country.

The nursing profession is teamwork that provides all kinds of health services in a balanced, accessible, continuous way with high quality and at the highest level. Nurses are indispensable and one of the essential elements of this team. Undoubtedly, nurses are the leaders or critical workers of multidisciplinary and interdisciplinary health teams in many countries. They also provide a wide range of services at all healthcare system levels and play an essential role in patient-centered care [7]. Nurses often have to move quickly between life and death, make critical decisions, and maintain the care they give to patients in unsafe environments [8]. In this case, in the literature, nursing is considered a profession with a heavy workload due to many negative factors arising from the working environment [9]. The nursing profession includes stress-related risk factors such as long-term work, time pressure, difficult or complex tasks, short rest breaks, monotony, and physically poor working conditions [10]. Standing up for a long time during treatment, sleeplessness during watches, nutritional irregularities, giving care to patients who are stressed due to their diseases, supporting patients and their relatives when necessary, patient care-related concerns, the obligation of establishing and maintaining good relations with the patient, the necessity of following the innovations in the field, irregular working hours, being obliged to empathize with the patients and their relatives in distress, intrateam conflicts, role ambiguity, and working environment-related stress cause pressure and strain for nurses [11]. Reasons such as this excessive workload in the nursing profession, the emotional stress experienced due to patient problems, and especially working with the shift system make working conditions difficult and increase the rate of making mistakes during nursing interventions [9, 12].

Mahaveerachartkul and Sooraksa [1] developed a scale that aims to determine whether nurses who have intense and tiring working hours and excessive workload perceive many stressful conditions in the working environment as a difficulty or an obstacle. It is thought that determining whether nurses perceive work stress as a difficulty or a hindrance with this study will guide health managers to make more real improvements in the hospital working environment. For example, it is believed that enabling nurses to better cope with difficulties and encounter fewer obstacles will increase their positive professional perceptions. This study aimed to adapt the “Challenge-Hindrance Demands Scale for The Nursing Profession” into Turkish.

## 2. Method

### 2.1. Design and Sample

This methodological study was carried out between March and May 2022. Nurses aged 18 years and over who could read and write in Turkish were included in the study. A survey prepared on docs.Google.com/forms was sent to the nurses via online tools (e-mail, WhatsApp, Facebook, and Instagram). In validity and reliability studies, ten times the number of items is considered sufficient to determine the sample size [13]. Considering that the scale consists of 14 items, a sample of at least 140 individuals was considered sufficient for the study. Data collected from 450 nurses were used in the study.

### 2.2. Data Collection Tools

The first part of the data collection form includes questions to determine sociodemographic characteristics. The second part consists of the items of the Challenge-Hindrance Demands Scale for The Nursing Profession.

#### 2.2.1. Demographic Data Form

The form consists of six questions regarding the sociodemographic characteristics of nurses, such as age, gender, marital status, educational status, duration of employment in the profession, and duration of employment in the unit they worked.

#### 2.2.2. Challenge-Hindrance Demands Scale for the Nursing Profession

The scale, which was developed by Mahaveerachartkul and Sooraksa [1], consists of 14 items and four subscales: Job Difficulty (items 1, 2, and 3), Time Requirements (items 4, 5, and 6), Time Requirements (items 7 and 8), and Intraorganizational Interaction (items 9, 10, 11, 12, 13, and 14).

### 2.3. Language Validity

Adapting a scale developed in a different language to a new language is defined as language validity [14]. In this study, five academicians who know English made the first translation of the original scale into Turkish. These translations were analyzed by an English language expert and transformed into a single text. The final text was back-translated into English by a translator who did not see the original questionnaire. By comparing the initial questionnaire with the translated text, whether the items had the same meaning was evaluated. It was decided that there was no difference in the purposes of the statements, and a pilot application was made with ten nurses to test the items' comprehensibility.

### 2.4. Content Validity

After the necessary corrections were made through the pilot application, the content validity was established. To test whether a scale is suitable for a new language, construct and content validity should be ensured [15]. Therefore, the content validity of the scale items was calculated using the Kendall W Test. Nurses who are experts in their fields were asked to evaluate the suitability of each scale item in terms of content and language on a range from 1 to 4 (1 point: inappropriate; 2 points: somewhat appropriate/requires revision; 3 points: right but requires small changes; 4 points: very reasonable). The percentage value of the answers reporting the appropriateness of each item was calculated with the scores given by each expert to the statements. When the responses of six experts were analyzed with Kendall's W test for the comprehensive items' comprehensibility, simplicity, and correlational validity, there was no statistical difference between the scale items and expert opinions (Kendall's *W* = 0.060; *p*=0.526 > 0.05).

### 2.5. Construct Validity

The construct validity of the scales used in the study was evaluated with exploratory factor analysis (EFA) and confirmatory factor analysis (CFA). There is no certainty about which fit indices are evaluated in model-data fit statistics calculated with confirmatory factor analysis. Values such as chi-square, CMIN/df, RMSEA, CFI, and TLI are presented [16]. Since the chi-square value, which indicates the fitness of data with the proposed model, is influenced by the sample size, its ratio to the degree of freedom provides more reliable results [17]. A CMIN/df value of less than two is considered a good fit, whereas a value of less than 5 refers to an acceptable fit. A root means square of error of approximation (RMSEA) value of 0.08 or less indicates an acceptable, and a value of 0.05 or less indicates a perfect fit [16]. The Tucker–Lewis Index (TLI) is generally between 0 and 1 but sometimes more significant than 1. A high TLI value indicates a good fit [17]. A comparative fit index (CFI) value greater than 0.95 is considered a good fit, and a value greater than 0.90 is considered acceptable [17]. Divergent and convergent validity was assessed by considering the average variance extracted (AVE), construct reliability (construct reliability; CR), and the square of the correlation values of the scales.

### 2.6. Data Analysis

The research data were analyzed in SPSS 26.0 and AMOS 24.0 statistical programs. The reliability of the scales used in the research was examined with the Cronbach alpha (CA) internal consistency coefficient. Descriptive findings were given in numbers, percentages, mean, and standard deviation values. A *p* value of <0.05 was considered statistically significant in the analyses.

### 2.7. Ethical Approvals

Permission was received from the owner of the scale via e-mail. Participation in the research was on an entirely voluntary basis. All information about the study was presented to the participants in a brief with the informed consent form. In addition, ethics committee approval dated 04.27.2022 and numbered 2022/364 was taken from the noninvasive clinical research ethics committee of Selçuk University Faculty of Health Sciences.

## 3. Results

This section presents descriptive findings of the participants, exploratory factor analysis, confirmatory factor analysis, reliability analysis, and correlation findings.

As seen in Table 1, the mean age of the participants was 34.80 ± 8.84. Of the participants, 325 (72.2%) were female, and 268 (59.6%) were married; 296 (65.8%) had a bachelor's degree. The mean duration of employment in the profession was 12.56 ± 9.35 years, and the mean duration of work in the unit was 5.86 ± 5.87 years.

Before EFA, corrected item-total correlations were examined for the research scales. The smallest value was 0.432 for the Challenge Demands Scale and 0.557 for the Hindrance Demand Scale. Therefore, the analyses were continued without removing any items. EFA analyses were performed with maximum likelihood estimation and Promax rotation methods.

According to the EFA findings of the Challenge Demands Scale, the KMO (0.919) and the Bartlett sphericity test (*χ*^2^ (78) = 4121.203; *p*=0.001) values were suitable for analysis. The analysis findings observed that the item “working under stressful time constraints” was included in a different subscale and removed from the study. The scale consists of 4 subscales and 13 items. The variance explained by Intraorganizational Interaction was 52.326%; the variance explained by Job Difficulty was 8.551%; the variance explained by Patient and Relative Management was 4.738; and the variance explained by Time Requirements was 2.958. The total explained variance was 68.572% (Table 2).

According to the EFA findings of the Hindrance Demands Scale, the KMO (0.947) value and the Bartlett sphericity test (*χ*^2^ (78) = 5263, 056; *p*=0.001) value were suitable for analysis. The analysis findings observed that the “heavy workload” was included in a different subscale and removed from the study. The scale consists of 4 subscales and 13 items. The variance explained by Intraorganizational Interaction was 61.68%; the variance explained by Job Difficulty was 4.352%; the variance explained by Time Requirements was 5.131%; and the variance explained by Patient and Relative Management was 3.261%. The total explained variance was 74.424% (Table 3).

**Figure 1:**
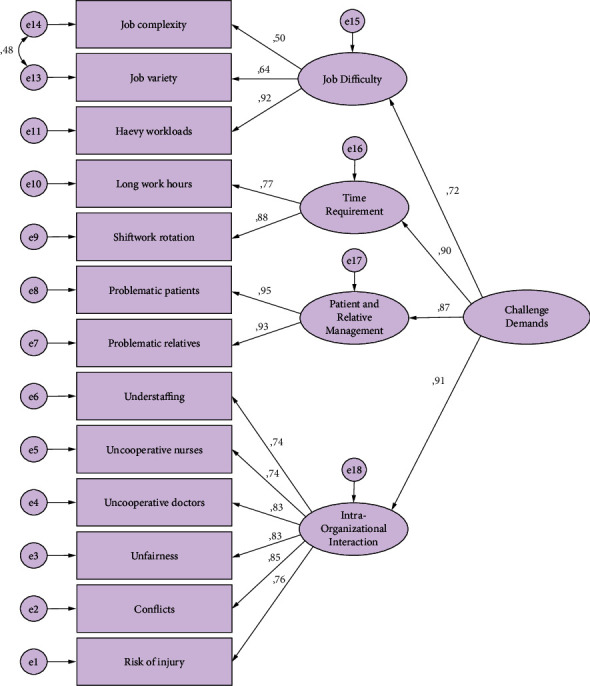
CFA model of the Challenge Demands Scale.

A second-level CFA was performed according to the scale structures created after EFA. When the CFA findings of the Challenge Demands Scale shown in Figure 1 were examined, it was seen that the fit indices were at an acceptable level (*χ*^2^ (60) = 223.912; *p*=0.001; CMIN/df = 3.732; CFI = 0.960; TLI = 0.948; RMSEA = 0.078; SRMR = 0.039). Factor loads were statistically significant (*p*=0.001). These findings showed that the scale is consistent with the data and is valid.

**Figure 2:**
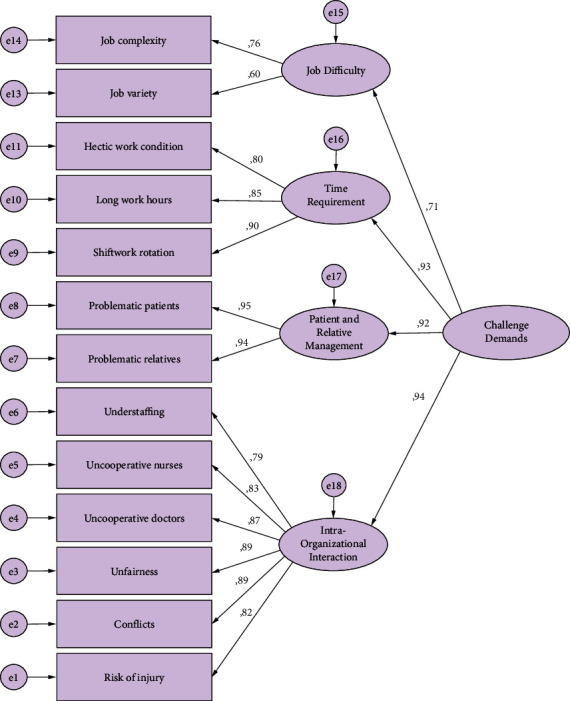
CFA model of the Hindrance Demands Scale.

When the CFA findings of the Hindrance Demands Scale shown in Figure 2 were examined, it was seen that the fit indices were at an acceptable level (*χ*^2^ (61) = 208.794; *p*=0.001; CMIN/df = 3.423; CFI = 0.972; TLI = 0.964; RMSEA = 0.073; SRMR = 0.032). Factor loads were statistically significant (*p*=0.001). These findings showed that the scale is consistent with the data and is valid.

The convergent and divergent validity of the scales was also examined. The AVE and CR values were 0.646 and 0.958 for the Challenge Demands Scale and 0.730 and 0.972 for the Hindrance Demands Scale, respectively. Since these values were more significant than 0.500 and 0.700, respectively, the scales had convergent validity. When the findings in Table 4 related to the factors were examined, it was seen that the rankings had convergent validity in terms of subscales. When the results were analyzed, it was seen that the AVE value for any two factors was higher than the square of the correlation value of these two factors. Thus, it was determined that the scales had divergent validity [18]. According to the CA values, the scales were found to be reliable.

## 4. Discussion

Stress can have devastating consequences by affecting individual well-being, behavior, and performance. Many studies focused on these stressors, emphasizing that minimizing stress will improve an individual's well-being [19–23]. However, stress can sometimes lead to positive consequences. Cavanaugh et al. [19] classified stressors as challenge stressors that can support an employee's goals and cause difficulties such as workload and time pressure and hindrance stressors that can prevent reaching a goal, such as a role ambiguity and role conflict. Both cases can cause stress. Stress resulting from challenge stressors was positively correlated with job satisfaction and negatively correlated with intention to leave the job [19]. This situation showed the opposite correlation in cases of stress caused by hindrance stressors. Similarly, in several studies, stress was addressed from different perspectives, and it was concluded that pressure had other effects on motivation, attitudes and behaviors, and performance [21, 23]. Accordingly, this study aimed to contribute to the national literature by establishing the Turkish validity and reliability of the Challenge-Hindrance Demands Scale for the Nursing Profession developed by Mahaveerachartkul and Sooraksa [1] to measure the stress, challenge, and hindrance levels that nurses face in their professional life. Each scale of the Challenge-Hindrance Demands Scale for the Nursing Profession has four subscales and 13 questions. These subscales are Job Difficulty, Time Requirements, Patient and Relative Management, and Intraorganizational Interaction.

After the content validity stage, EFA and CFA were performed to test the construct validity. Before the factor analysis, KMO and Bartlett sphericity tests were performed to test the sample size and its suitability for factor analysis. The KMO coefficient was 0.94 for the Hindrance Demands Scale and 0.91 for the Challenge Demands Scale, indicating that the sample size was pretty sufficient [24]. The results of the Bartlett sphericity test were significant, indicating that the scale is suitable for factor analysis.

The total variance ratio should be above 50% for multidimensional scales [17]. This value was determined as 0.646 for the Challenge Demands Scale and 0.730 for the Hindrance Demands Scale. As a result of exploratory factor analysis, it was determined that the scales preserved their original four-factor structure.

It was aimed to test the structure created after EFA with confirmatory factor analysis. In adaptation studies, CFA is used to determine whether the original design is confirmed with newly collected data [17]. With this analysis, the scale and factors are re-assessed. The process starts with a set of observed variables in confirmatory factor analysis like in EFA. The relationship between the variables is explained by using a smaller number under the factors [25]. As a result of the analysis, the measurement model established to confirm the 14 item structures was approved with 13 items and four subscales. To improve the goodness of fit values in the confirmatory factor analysis of the Challenge Demands Scale, covariance analysis was performed between the first and second items in the same factor. As a result of covariance, the calculation was made again, and the goodness of fit index values was recalculated. When the CFA findings of the Challenge Demands Scale were examined to determine whether the original structure of the scale was confirmed in Turkish participants, it was seen that the fit indices were at an acceptable level (*χ*^2^ (60) = 223.912; *p*=0.001; CMIN/df = 3.732; CFI = 0.960; TLI = 0.948; RMSEA = 0.078; SRMR = 0.039). When the CFA findings of the Hindrance Demands Scale were examined, similarly, it was seen that the fit indices were at an acceptable level (*χ*^2^ (61) = 208.794; *p*=0.001; CMIN/df = 3.423; CFI = 0.972; TLI = 0.964; RMSEA = 0.073; SRMR = 0.032). Factor loads were statistically significant (*p*=0.001). These findings show that the scale is valid and consistent with the data.

The Cronbach alpha reliability coefficient and item-total correlation were used to calculate the scale's reliability. It is recommended that the Cronbach alpha reliability coefficient should be close to 1 and values greater than 0.6 should be taken into account [17, 26]. According to the reliability analysis results, the Cronbach alpha coefficients for the Job Difficulty, Time Requirements, Patient and Relative Management, and Intraorganizational Interaction subscales were 0.794, 0.809, 0.936, and 0.908, respectively, for the Challenge Demands Scale and 0.756, 0.885, 0.944, and 0.938, respectively, for the Hindrance Demands Scale. It was seen that all four subscales had good reliability for both scales. These values in the English and Turkish versions of the scale are similar. These findings showed that the Turkish version of the scale also has a high internal consistency.

## 5. Conclusion

In conclusion, it has been determined that the developed and validated Challenge-Hindrance Demands Scale for the Nursing Profession can be used in studies to measure the stress, challenge, and hindrance levels that nurses encounter in their working life in Turkey. The results confirmed the 4-factor structure of the scales. The Cronbach internal consistency coefficient and item-total correlation of the scale were considered sufficient.

## Figures and Tables

**Table 1:** Descriptive findings regarding participants' demographic characteristics.

		*n*	%
Age	Mean ± SD (34.80 ± 8.84)		

Gender	Female	325	72.2
	Male	125	27.8

Marital status	Married	268	59.6
	Single	182	40.4

Education	High school	25	5.6
	Associate	56	12.4
	Bachelor's	296	65.8
	Postgraduate	73	16.2

Duration of employment in the profession	Mean ± SD (12.56 ± 9.35)		
Duration of employment in the unit	Mean ± SD (5.86 ± 5.87)		

**Table 2:** Exploratory factor analysis findings of the Challenge Demands Scale.

Challenge Demands Scale	F1	F2	F3	F4
**Complex or difficult work**	−0.009	**0.801**	−0.015	−0.088
**Responsibility of multiple tasks**	−0.048	**0.864**	0.014	0.015
**Heavy workload**	0.177	**0.442**	−0.025	0.295
**Working more than normal daily working hours**	−0.079	0.031	0.033	**0.854**
**Irregular work schedule**	0.258	−0.115	0.073	**0.641**
**Working with patients (overly demanding, nervous, uncooperative, etc.) who increase work stress**	0.094	0.010	**0.861**	0.003
**Working with patient relatives (over-demanding, nervous, uncooperative, etc.,) who increase work stress**	0.026	−0.024	**0.887**	0.057
**Lack of staff in the unit**	**0.434**	0.107	0.232	0.069
**Working with noncompetent nurses in the unit**	**0.638**	0.111	0.042	−0.005
**Working with doctors who increase work stress (overly demanding, closed to new ideas, uncooperative, etc.)**	**0.771**	0.022	0.146	−0.093
**Unfair treatment at work (e.g., by the supervisor or coworkers)**	**0.915**	−0.048	0.066	−0.125
**Conflict within or between departments**	**0.945**	−0.005	−0.149	0.057
**Risk of injury or illness**	**0.732**	−0.089	−0.033	0.137
Eigenvalues	6.802	1.112	0.616	0.385
Explained variance (%)	52.326	8.551	4.738	2.958
Total explained variance (%)	52.326	60.876	65.614	68.572

**Table 3:** Exploratory factor analysis findings of the Hindrance Demands Scale.

Hindrance Demands Scale	F1	F2	F3	F4
**Complex or difficult work**	0.088	**0.712**	−0.049	−0.006
**Responsibility of multiple tasks**	−0.069	**0.851**	0.018	0.017
**Working under stressful time constraints**	0.087	0.292	**0.513**	0.000
**Working more than normal daily working hours**	0.028	0.095	**0.753**	0.009
**Irregular work schedule**	0.134	−0.125	**0.900**	0.000
**Working with patients (overly demanding, nervous, uncooperative, etc.,) who increase work stress**	0.162	0.013	0.104	**0.694**
**Working with patient relatives (over-demanding, nervous, uncooperative, etc.,) who increase work stress**	0.052	0.012	−0.036	**0.959**
**Lack of staff in the unit**	**0.428**	0.156	0.106	0.187
**Working with noncompetent nurses in the unit**	**0.613**	−0.068	0.162	0.147
**Working with doctors who increase work stress (overly demanding, closed to new ideas, uncooperative, etc.)**	**0.827**	0.103	0.018	−0.044
**Unfair treatment at work (e.g., by the supervisor or coworkers)**	**0.943**	−0.067	0.012	−0.006
**Conflict within or between departments**	**0.953**	0.055	−0.080	−0.024
**Risk of injury or illness**	**0.605**	−0.052	0.219	0.074
Eigenvalues	8.018	0.566	0.667	0.424
Explained variance (%)	61.680	4.352	5.131	3.261
Total explained variance (%)	61.680	66.032	71.163	74.424

**Table 4:** Correlation findings regarding scales.

Subscale	1	2	3	4	5	6	7	8
1. CDS JD	—	0.254	0.209	0.225	0.028	0.000	0.001	0.000
2. CDS TR	0.504^*∗∗*^	—	0.447	0.459	0.007	0.026	0.021	0.020
3. CDS PRM	0.458^*∗∗*^	0.669^*∗∗*^	—	0.535	0.004	0.029	0.052	0.031
4. CDS IOI	0.475^*∗∗*^	0.678^*∗∗*^	0.732^*∗∗*^	—	0.001	0.039	0.030	0.046
5. HDM JD	−0.170^*∗∗*^	−0.088	−0.069	−0.043	—	0.320	0.288	0.286
6. HDM TR	0.015	−0.162^*∗∗*^	−0.171^*∗∗*^	−0.199^*∗∗*^	0.566^*∗∗*^	—	0.582	0.643
7. HDM PRM	0.032	−0.146^*∗∗*^	−0.230^*∗∗*^	−0.174^*∗∗*^	0.537^*∗∗*^	0.763^*∗∗*^	—	0.669
8. HDM IOI	0.027	−0.142^*∗∗*^	−0.178^*∗∗*^	−0.215^*∗∗*^	0.535^*∗∗*^	0.802^*∗∗*^	0.818^*∗∗*^	—
Mean	7.94	3.88	4.11	11.62	5.29	9.31	6.39	19.22
Std. deviation	2.94	1.95	2.19	5.41	2.18	3.57	2.61	6.97
AVE	0.500	0.687	0.878	0.629	0.608	0.722	0.894	0.720
CR	0.738	0.813	0.935	0.910	0.756	0.886	0.944	0.939
CA	0.794	0.809	0.936	0.908	0.756	0.885	0.944	0.938

CDS JD = Challenge Demands Scale Job Difficulty; CDS TR = Challenge Demands Scale Time Requirements; CDS PRM = Challenge Demands Scale Patient and Relative Management; CDS IOI = Challenge Demands Scale Intraorganizational Interaction; HDS JD = Hindrance Demands Scale Job Difficulty; HDS TR = Hindrance Demands Scale Time Requirements; HDS PRM = Hindrance Demands Scale Patient and Relative Management; HDS IOI = Hindrance Demands Scale Intraorganizational Interaction; AVE = average variance extracted; CR = construct reliability; CA = Cronbach alpha. Values below the diagonal indicate the correlation between factors, and values above the diagonal indicate the square of the correlation value. ^*∗∗*^*p*=0.001.

## Data Availability

The data that support the finding of this study are available upon request from the corresponding author.
